# Replacement of the Common Chromium Source CrCl_3_(thf)_3_ with Well-Defined [CrCl_2_(μ-Cl)(thf)_2_]_2_

**DOI:** 10.3390/molecules26041167

**Published:** 2021-02-22

**Authors:** Dong Geun Lee, Jun Won Baek, Jung Hyun Lee, Hyun Ju Lee, Yeong Hyun Seo, Junseong Lee, Chong Gu Lee, Bun Yeoul Lee

**Affiliations:** 1 Department of Molecular Science and Technology, Ajou University, 206 Worldcup-ro, Yeongtong-gu, Suwon 16499, Korea; itl1096@ajou.ac.kr (D.G.L.); btw91@ajou.ac.kr (J.W.B.); moa0811@hanmail.net (J.H.L.); hjulee4639@ajou.ac.kr (H.J.L.); tdg0730@ajou.ac.kr (Y.H.S.); 2 Department of Chemistry, Chonnam National University, 77 Yongbong-ro, Buk-gu, Gwangju 61186, Korea; leespy@chonnam.ac.kr; 3 Precious Catalysts Inc. 201, Duryu-gil, Angangeup, Gyeongju 38029, Korea; cglee@s-pci.com

**Keywords:** chromium compound, CrCl_3_(thf)_3_, [CrCl_2_(μ-Cl)(thf)_2_]_2_, ethylene tetramerization catalyst

## Abstract

CrCl_3_(thf)_3_ is a common starting material in the synthesis of organometallic and coordination compounds of Cr. Deposited as an irregular solid with no possibility of recrystallization, it is not a purity guaranteed chemical, causing problems in some cases. In this work, we disclose a well-defined form of the THF adduct of CrCl_3_ ([CrCl_2_(μ-Cl)(thf)_2_]_2_), a crystalline solid, that enables structure determination by X-ray crystallography. The EA data and XRD pattern of the bulk agreed with the revealed structure. Moreover, its preparation procedure is facile: evacuation of CrCl_3_·6H_2_O at 100 °C, treatment with 6 equivalents of Me_3_SiCl in a minimal amount of THF, and crystallization from CH_2_Cl_2_. The ethylene tetramerization catalyst [iPrN{P(C_6_H_4_-*p*-Si(nBu)_3_)_2_}_2_CrCl_2_]^+^[B(C_6_F_5_)_4_]^−^ prepared using well-defined [CrCl_2_(μ-Cl)(thf)_2_]_2_ as a starting material exhibited a reliably high activity (6600 kg/g-Cr/h; 1-octene selectivity at 40 °C, 75%), while that of the one prepared using the impure CrCl_3_(thf)_3_ was inconsistent and relatively low (~3000 kg/g-Cr/h). By using well-defined [CrCl_2_(μ-Cl)(thf)_2_]_2_ as a Cr source, single crystals of [(CH_3_CN)_4_CrCl_2_]^+^[B(C_6_F_5_)_4_]^−^ and [{Et(Cl)Al(N(iPr)_2_)_2_}Cr(μ-Cl)]_2_ were obtained, allowing structure determination by X-ray crystallography, which had been unsuccessful when the previously known CrCl_3_(thf)_3_ was used as the Cr source.

## 1. Introduction

CrCl_3_(thf)_3_ has been widely used as a starting material for the synthesis of organometallic and coordination compounds of Cr [1,2,3,4,5,6,7,8,9,10]. Its preparation method was reported 60 years ago; anhydrous CrCl_3_, which is insoluble in organic solvents as well as in water, was extracted with THF by the aid of Zn dust using Soxhlet apparatus [11,12,13]. However, anhydrous CrCl_3_ is prepared by hazardous and not-facile process (treatment of Cr_2_O_3_·*x*H_2_O with CCl_4_ at 630 °C leads to the generation of extremely toxic phosgene gas), and thus, is expensive [14,15]. Another method was reported in which CrCl_3_·6H_2_O was reacted with excess thionyl chloride (S(O)Cl_2_) to generate anhydrous CrCl_3_. In this process, a different form of hygroscopic CrCl_3_ was generated, from which CrCl_3_(thf)_3_ could be immediately obtained after adding THF [16,17,18]. The most attractive and convenient method is reacting CrCl_3_·6H_2_O with excess Me_3_SiCl in THF, resulting in the deposition of CrCl_3_(thf)_3_ along with the generation of byproducts, HCl and Me_3_SiOSiMe_3_ (CrCl_3_·6H_2_O + 12 Me_3_SiCl → CrCl_3_(thf)_3_ + 12 HCl + 6 Me_3_SiOSiMe_3_) [19]. Excess Me_3_SiCl (~60 equiv./Cr) needs to be added to obtain a completely anhydrous form of CrCl_3_(thf)_3_ [20]. Otherwise (e.g., when 25 equiv. of Me_3_SiCl/Cr was added according to the literature), hydrated CrCl_3_(thf)_2_(H_2_O) is obtained, which was erroneously sold as CrCl_3_(thf)_3_ in the past [20,21].

**Scheme 1:**
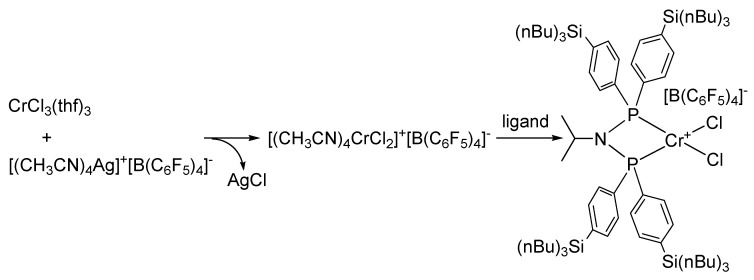
Preparation of extremely active ethylene tetramerization catalyst.

Sasol disclosed a catalyst system composed of a Cr source, iPrN(PPh_2_)_2_ ligand, and methylaluminoxane (MAO), which produces mainly 1-octene through ethylene tetramerization [22,23,24]. CrCl_3_(thf)_3_ has been actively used as a component of the ethylene tetramerization catalyst system [25,26,27,28,29,30,31,32,33,34]. We also developed a very efficient catalyst for ethylene tetramerization (Scheme 1) [35,36,37]. The catalyst shows extremely high activity (~5000 kg/g-Cr/h) and is advantageous over other reported systems in that it works with an inexpensive activator, iBu_3_Al, avoiding the use of expensive MAO in excess (A1/Cr = 300–500) [38,39,40,41,42,43]. During the course of the studies, we had a problem in reproducing such an extremely high activity and eventually found that the Cr source CrCl_3_(thf)_3_ used in the preparation was the cause of this problem. Although the structure of CrCl_3_(thf)_3_ was revealed by X-ray crystallography, using a single crystal selected from the batch in the Soxhlet extraction process, it does not redeposit as good-quality crystals when redissolved in CH_2_Cl_2_ [44,45]. Structure elucidated by X-ray crystallography does not guarantee the purity of the bulk and we questioned the purity of the common Cr source CrCl_3_(thf)_3_. In fact, it was reported as an “irregular violet solid” which may mean that it is not a pure CrCl_3_(thf)_3_ but a mixture containing other forms of THF adduct of CrCl_3_ (e.g., μ2-Cl bridged multinuclear species) [46]. Elemental analysis (EA) data for Cr and Cl have been reported, but common carbon and hydrogen data were missing. CrCl_3_(thf)_3_ is paramagnetic; thus, it cannot be subjected to structural analysis using ^1^H and ^13^C NMR spectroscopy. We also previously found that the composition (as well as the structure) of the commercial source of (2-ethylhexanoate)_3_Cr, another type of important paramagnetic Cr(III) complex that is commercially used as a component in the Phillips ethylene trimerization catalyst [47]. is erroneous [21,48]. By correcting the structure to (2-ethylhexanoate)_2_CrOH, the catalytic performance was consistent and, moreover, significantly improved.

## 2. Results and Discussion

CrCl_3_(thf)_3_ was commercially available from Aldrich (St. Louis, MO, USA), but we prepared it to ensure complete dryness by reacting CrCl_3_·6H_2_O with excess Me_3_SiCl (60 equiv.) [20]. We modified the procedure as follows: CrCl_3_·6H_2_O was evacuated for ~6 h before the treatment with Me_3_SiCl. The catalyst [iPrN{P(C_6_H_4_-*p*-Si(nBu)_3_)_2_}_2_CrCl_2_]^+^[B(C_6_F_5_)_4_]^−^, prepared according to Scheme 1 using CrCl_3_(thf)_3_, which was prepared via the evacuation step, provided extremely high activity (~5000 kg/g-Cr/h). In contrast, the catalyst prepared using either the commercial source of CrCl_3_(thf)_3_ or the one prepared according to the reported method without the evacuation step did not exhibit such a high activity (~3000 kg/g-Cr/h), and there was some amount of methylcyclohexane-insoluble fraction when ligand iPrN{P(C_6_H_4_-*p*-Si(nBu)_3_)_2_}_2_ was reacted with [(CH_3_CN)_4_CrCl_2_]^+^[B(C_6_F_5_)_4_]^−^. The insoluble fraction was negligible when CrCl_3_(thf)_3_, which was prepared via the evacuation step, was used as the starting material.

**Scheme 2:**
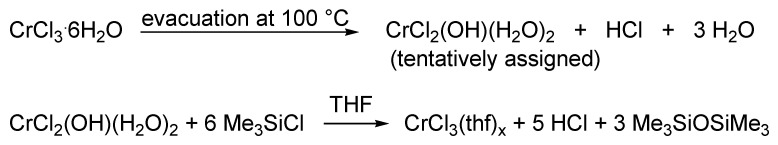
Modified preparation method for THF adduct of CrCl_3_.

We investigated what happened in the evacuation step. When the crystalline form of CrCl_3_·6H_2_O (10 g, 37.5 mmol) was evacuated at 100 °C, the color gradually changed from dark green to light green, light gray, and finally light purple with gradual loss of weight. Finally, an amorphous powder was obtained, and the weight loss converged to 36% (6.39 g remaining). Further weight reduction was minimal when the evacuation time was increased. It was confirmed with litmus paper that the removed volatile component was not pure water but acidic, presumably containing HCl. From these observations, the remaining Cr compound was tentatively considered as CrCl_2_(OH)(H_2_O)_2_; 34% weight loss is expected for the transformation of CrCl_3_·6H_2_O to CrCl_2_(OH)(H_2_O)_2_ (Scheme 2). The structure of CrCl_3_·6H_2_O was reported to be [CrCl_2_(H_2_O)_4_]Cl·2H_2_O, and the action of heat under vacuum could liberate outer-sphere Cl^−^ (as HCl with a proton in an inner-sphere water) and three water molecules (two from the outer sphere and one from the inner sphere), consequently leaving CrCl_2_(OH)(H_2_O)_2_ (possibly, [CrCl_2_(H_2_O)_2_(μ-OH)]_2_ adopting an octahedral structure) in the reaction pot.

Treatment of CrCl_2_(OH)(H_2_O)_2_, after dissolving in THF, with Me_3_SiCl resulted in the precipitation of a violet solid, which was confirmed by infrared spectroscopy to be completely anhydrous. Because a large portion of H_2_O in CrCl_3_·6H_2_O was removed at the evacuation step, 6.0 equiv. Me_3_SiCl (equivalent amount that needed for the transformation of CrCl_2_(OH)(H_2_O)_2_ to CrCl_3_(thf)_x_, Scheme 2) was added instead of 60 equiv./Cr, and accordingly, the amount of THF could be minimized (36 mL/10 g—CrCl_3_·6H_2_O). The color and shape of the precipitated solid (irregular violet solid) were almost identical to that of the commercial source of CrCl_3_(thf)_3_ and the one prepared without the evacuation step. However, as aforementioned, the catalyst performance was significantly better when using CrCl_3_(thf)_3_ prepared via the evacuation step. The product of the reaction between [(CH_3_CN)_4_Ag]^+^[B(C_6_F_5_)_4_]^−^ and CrCl_3_(thf)_3_ (i.e., [(CH_3_CN)_4_CrCl_2_]^+^[B(C_6_F_5_)_4_]^−^) differed depending on the source of CrCl_3_(thf)_3_. Although [(CH_3_CN)_4_CrCl_2_]^+^[B(C_6_F_5_)_4_]^−^ was isolated by precipitation in CH_3_CN at −30 °C, the precipitate was not composed of good-quality crystals; thus, X-ray crystallography could not be used for characterization. We crosschecked the tentatively assigned structure of [(CH_3_CN)_4_CrCl_2_]^+^[B(C_6_F_5_)_4_]^−^ by counting the number of CH_3_CN molecules per each Cr atom through the analysis of integration values in the ^1^H-NMR spectra recorded using THF-d_8_ with 9-methylanthracene as an internal standard. For the product obtained using CrCl_3_(thf)_3_ prepared via the evacuation step, the number of CH_3_CN per Cr was in good agreement with the expected value of 4 (4.1, 3.8, or 4.0). In contrast, the number deviated from 4 (6.5, 6.1, 5.3, or 6.2) when either the commercial source of CrCl_3_(thf)_3_ or the one prepared without the evacuation step was used.

**Figure 1:**
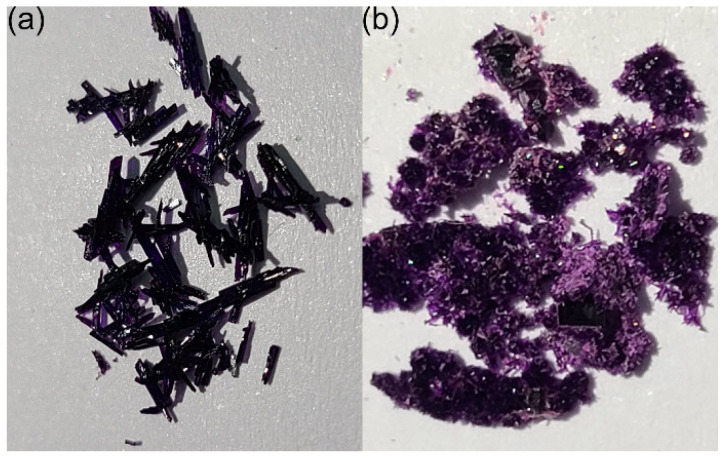
Macroscopic views of crystals of [CrCl_2_(μ-Cl)(thf)_2_]_2_ (**a**) and irregular solid known as CrCl_3_(thf)_3_ (**b**).

**Figure 2:**
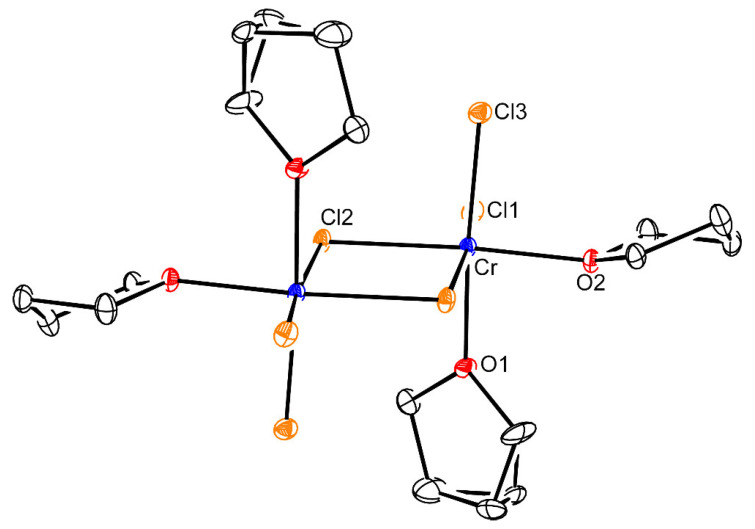
X-ray structure of [CrCl_2_(μ-Cl)(thf)_2_]_2_. Selected bond distances (Å) and angles (°): Cr-Cl1, 2.2773(12); Cr-Cl2, 2.3622(12); Cr-Cl3, 2.2885(11); Cr-O1, 2.045(3); Cr-O2, 2.018(3); C-O1-C, 109.5(3); C-O1-Cr, 125.5(2); C-O1-Cr, 125.0(2); C-O2-C, 108.7(3); C-O2-Cr, 121.5(2); and C-O2-Cr, 122.6(2).

The most striking difference between the two samples was that good-quality crystals were deposited in a CH_2_Cl_2_ solution of CrCl_3_(thf)_3_ that was prepared via the evacuation step, whereas an irregular solid was deposited when either the commercial source of CrCl_3_(thf)_3_ or the one prepared without the evacuation step was dissolved in CH_2_Cl_2_ (Figure 1). X-ray crystallography studies revealed that the deposited crystals were a Cl-bridged dinuclear complex [CrCl_2_(μ-Cl)(thf)_2_]_2_ (Figure 2). The Cr atom adopted an octahedral structure, with two THFs being in the cis-position. The bond distances between Cr and terminal-Cl (2.277 and 2.289 Å) were shorter than that between Cr and bridge-Cl (2.362 Å) as well as the Cr–Cl distances (3.299, 3.318, and 3.352 Å) observed for *mer*-CrCl_3_(thf)_3_, whose structure has been previously revealed using a single crystal obtained from the reaction pot of Cr(CH_2_SiMe_3_)_4_ and HCl in THF (CCDC# 983659) [20]. The distance between Cr and O^THF^ situated trans to terminal-Cl was greater than that between Cr and O^THF^ situated trans to bridge-Cl (2.045 vs. 2.018 Å). The O^THF^ atom situated trans to terminal-Cl adopted perfect trigonal planar geometry (sum of bond angle around O1 atom, 360.0°), indicating π-donation from O to Cr through adopting sp^2^-hybridization. The other O^THF^ atom situated trans to bridge-Cl deviated from a perfect trigonal planar geometry (sum of bond angle around O1 atom, 352.8°).

X-ray crystallography data do not guarantee the purity of the bulk. The EA data of C were roughly in agreement with the values calculated for the revealed structure [CrCl_2_(μ-Cl)(thf)_2_]_2_ (31.8%), although the measured values were not always within the required range of 31.8% ± 0.4% (30.7%, 30.9%, 30.4%, 31.0%, 30.5%, 30.2%, 30.4%, 30.9%, and 31.2%). In contrast, the EA data of C measured for either the commercial source of CrCl_3_(thf)_3_ or the one prepared without the evacuation step deviated substantially from the value calculated for CrCl_3_(thf)_3_ (38.5%). The data fluctuated severely by variation in sampling points (32.4%, 26.5%, 34.1%, 38.1%, and 33.8% for a bottle of purchased sample; 34.2%, 36.7%, 37.1%, 35.3%, and 34.1% for another bottle of purchased sample; 31.3%, 30.5%, 37.0%, 33.6%, and 33.9% for the sample prepared without the evacuation step). Before recrystallization, the sample prepared via the evacuation step also showed fluctuating C content by varying the sampling point, and the C content agreed neither with the structure of CrCl_3_(thf)_3_ nor the structure of [CrCl_2_(μ-Cl)(thf)_2_]_2_ (35.3%, 34.3%, 32.5%, 34.2%, and 32.4%). Though the carbon contents and the XRD pattern are inconsistent with the formula of the commercial source CrCl_3_(THF)_3_, the Cr content measured by ICP-OES (Inductively Coupled Plasma Optical Emission Spectroscopy) and the Cl content measured by gravimetric analysis after treatment of AgNO_3_ to aqueous solution of CrCl_3_(THF)_3_ were not severely deviated from the values calculated for the formula CrCl_3_(THF)_3_ (Calcd. Cr 13.88, Cl 28.38. Found: Cr 14.04, Cl 29.09%). The Cr content measured for the prepared [CrCl_2_(μ-Cl)(thf)_2_]_2_ agreed accurately with the calculated value while the measured Cl content was slightly deviated (Calcd. Cr 17.19, Cl 35.15. Found: Cr 17.18, Cl 34.58%).

**Figure 3:**
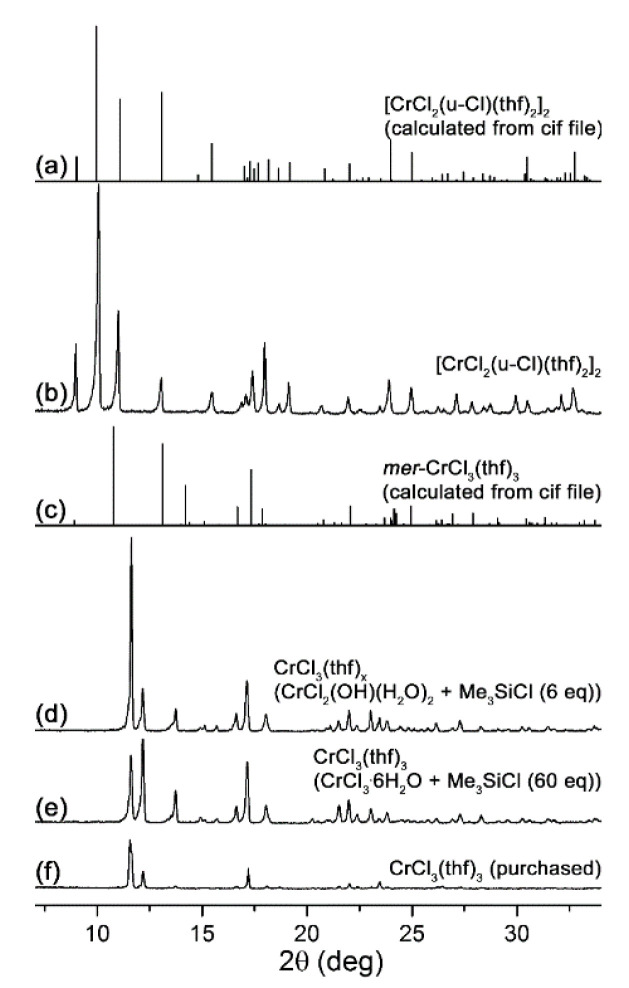
X-ray powder diffraction (XRD) patterns of [CrCl_2_(μ-Cl)(thf)_2_]_2_ calculated from cif file (**a**) and measured (**b**) and those of CrCl_3_(thf)_3_ calculated from cif file (**c**), obtained from the reaction ‘CrCl_2_(OH)(H_2_O)_2_ + Me_3_SiCl (6 eq)’ (**d**), obtained from the reaction ‘CrCl_3_·6H_2_O + Me_3_SiCl (60 eq)’ (**e**), and purchased (**f**).

**Figure 4:**
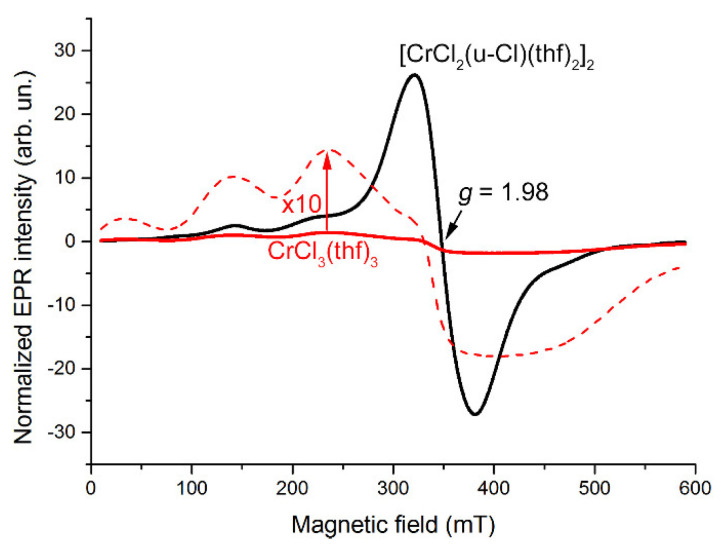
EPR spectra of [CrCl_2_(μ-Cl)(thf)_2_]_2_ and the commercial CrCl_3_(thf)_3_.

The X-ray powder diffraction (XRD) pattern observed for bulk [CrCl_2_(μ-Cl)(thf)_2_]_2_ isolated via the recrystallization procedure agreed well with single-crystal X-ray crystallography data (cif file) (Figure 3a vs. Figure 3b), indicating that the majority of deposited solid had a structure of [CrCl_2_(μ-Cl)(thf)_2_]_2_. In contrast, the XRD pattern of the purchased CrCl_3_(thf)_3_ or the one prepared by the reported method without the evacuation step did not agree with single-crystal X-ray crystallography data of *mer*-CrCl_3_(thf)_3_ (Figure 3c vs. Figure 3e,f). This observation, along with the mismatched EA data, creates uncertainty about the structure and composition of the purchased CrCl_3_(thf)_3_ as well as of the one prepared by the reported method. The solid deposited in the reaction pot of CrCl_2_(OH)(H_2_O)_2_ and Me_3_SiCl (6 eq) showed a large signal at 2θ = 11.6° along with the signals observed for the CrCl_3_(thf)_3_ samples (Figure 3d). The electron paramagnetic resonance (EPR) spectrum of [CrCl_2_(μ-Cl)(thf)_2_]_2_ showed a strong signal at g = 1.98, while relatively weak signals were observed for the CrCl_3_(thf)_3_ samples; in addition, the signal patterns were completely different for the two samples (Figure 4). In thermogravimetry analysis (TGA) of CrCl_3_(thf)_3_, roughly four stages of the weight loss were observed (30%, 50%, 60%, and 84% at 170 °C, 215 °C, 300 °C, and 1000 °C, respectively), while three stages of the weight loss were roughly observed for [CrCl_2_(μ-Cl)(thf)_2_]_2_ (35%, 53%, and 73% at 220 °C, 310 °C, and 1000 °C, respectively).

**Figure 5:**
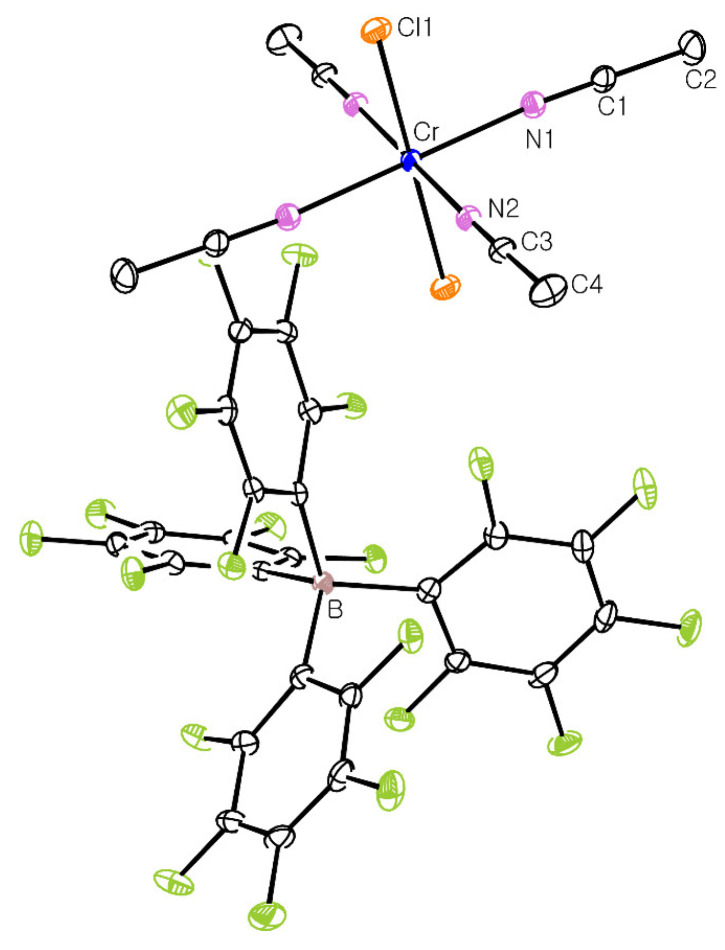
X-ray structure of [(CH_3_CN)_4_CrCl_2_]^+^[B(C_6_F_5_)_4_]^−^. Selected bond distances (Å) and angles (°): Cr-N1, 2.025(3); Cr-N2, 2.018(3); Cr-Cl1, 2.2804(9); Cr-N2-C, 178.8(3); and Cr-N1-C, 175.5(3).

Many attempts to grow single crystals of [(CH_3_CN)_4_CrCl_2_]^+^[B(C_6_F_5_)_4_]^−^ have been unsuccessful when [(CH_3_CN)_4_Ag]^+^[B(C_6_F_5_)_4_]^−^ was reacted with the purchased CrCl_3_(thf)_3_ or the one prepared through the reaction of CrCl_3_·6H_2_O and excess Me_3_SiCl (60 eq) without the evacuation step (Scheme 1) [38]. However, single crystals suitable for X-ray crystallography could be obtained when [CrCl_2_(μ-Cl)(thf)_2_]_2_ was reacted with [(CH_3_CN)_4_Ag]^+^[B(C_6_F_5_)_4_]^−^. The structure determined by X-ray crystallography is shown in Figure 5. By reacting well-defined [(CH_3_CN)_4_CrCl_2_]^+^[B(C_6_F_5_)_4_]^−^ with a iPrN{P(C_6_H_4_-*p*-Si(nBu)_3_)_2_ ligand [37], a high-quality Cr complex [iPrN{P(C_6_H_4_-*p*-Si(nBu)_3_)_2_}_2_CrCl_2_]^+^[B(C_6_F_5_)_4_]^−^ was produced, which showed extremely high activity in the ethylene tetramerization reaction (activity: 6600 kg/g-Cr/h; selectivity: 1-octene—74.7%, 1-hexene—7.5%, cyclic-C6—3.8%, C10+—13.8%, PE—0.2%; reaction conditions: 34 bar ethylene, 235 mL methylcyclohexane, 0.45 mmol Cr catalyst, 90 mmol (iBu)_3_Al, 40 °C, 30 min). The Cr complex [iPrN{P(C_6_H_4_-*p*-Si(nBu)_3_)_2_}_2_CrCl_2_]^+^[B(C_6_F_5_)_4_]^−^ is highly soluble in methylcyclohexane, which is advantageous in handling and operating the tetramerization reaction, and there is no way for purification, necessitating a high-quality Cr precursor [(CH_3_CN)_4_CrCl_2_]^+^[B(C_6_F_5_)_4_]^−^ as well as high-quality ligand iPrN{P(C_6_H_4_-*p*-Si(nBu)_3_)_2_}_2_ to ensure consistently high activity.

**Figure 6:**
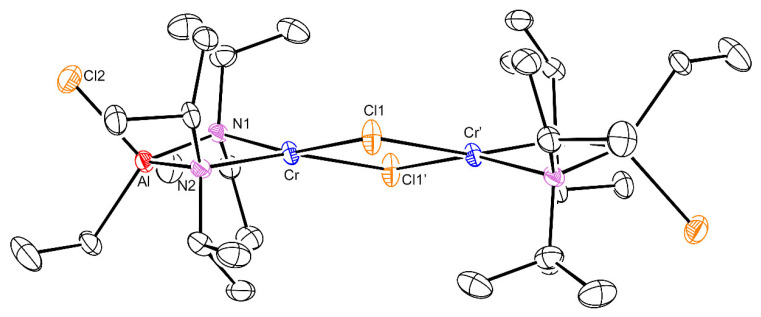
X-ray structure of [{Et(Cl)Al(N(iPr)_2_)_2_}Cr(μ-Cl)]_2_. Selected bond distances (Å) and angles (°): Cr-Cl1, 2.4212; Cr-N1, 2.141(6); Cr-N2, 2.119(6); Al-N1, 1.925(6); Al-N2, 1.953(6); Al-Cl2, 2.163(3); Cr--Cr’, 3.578(2); N1-Cr-N2, 84.3(2); Cl-Cr-Cl’, 83.96(8); N1-Al-N2, 95.0(3); and angle between N1-Cr-N2 and Cl-Cr-Cl’ planes, 25.1(1).

In previous studies, we encountered a similar problem possibly caused by the use of impure CrCl_3_(thf)_3_. In the reaction of Li^+^[Et_2_Al(N(iPr)_2_)_2_]^−^ and CrCl_3_(thf)_3_, solids were isolated; however, their structure could not be determined by X-ray crystallography. The product was tentatively considered to be [Et_2_Al(N(iPr)_2_)_2_]CrCl_2_ [48]. However, single crystals were obtained when well-defined [CrCl_2_(μ-Cl)(thf)_2_]_2_ replaced the impure CrCl_3_(thf)_3_, enabling structure determination by X-ray crystallography. The complex isolated from the reaction of Li^+^[Et_2_Al(N(iPr)_2_)_2_]^−^ and CrCl_3_(thf)_3_ was not a Cr(III) complex [Et_2_Al(N(iPr)_2_)_2_]CrCl_2_ but a Cr(II) complex [{Et(Cl)Al(N(iPr)_2_)_2_}Cr(μ-Cl)]_2_ (Figure 6).

## 3. Materials and Methods

### 3.1. General Remarks

All manipulations were performed in an inert atmosphere using a standard glove box and Schlenk techniques. CH_2_Cl_2_ and CH_3_CN were stirred over CaH_2_ and transferred to a reservoir under vacuum. Toluene, hexane, and THF were distilled from benzophenone ketyl. EAs were carried out at the Analytical Center, Ajou University. CW X-band EPR spectra were collected at room temperature on an EMX plus 6/1 spectrometer (Bruker, Billerica, MA, USA) with experimental parameters of 1 mW microwave power, 10 G modulation amplitude, and 3 scans at KBSI Western Seoul Center Korea. HP-XRD data were obtained on a D/max-2500V/PC (Rigaku, Akishima, Japan) equipped with a Cu Kα radiation source (λ = 0.15418 nm).

### 3.2. [CrCl_2_(μ-Cl)(thf)_2_]_2_

A Schlenk flask containing crystalline solid CrCl_3_·6H_2_O (10.0 g, 37.5 mmol) was immersed in an oil bath at a temperature of 40 °C and then evacuated under full vacuum for 1 h. Under evacuation, the bath temperature was raised to 100 °C over an hour and then maintained at 100 °C for 4 h. As volatiles were removed, the crystalline solid changed to an amorphous powder and the color of the solid gradually changed from dark green to light green, light gray, and finally light purple. The weight reduced from 10.0 to 6.39 g by the removal of volatiles. Cold THF (32 g, −30 °C) was added to dissolve the remaining solid. By dissolution, heat was generated, and a dark-purple solution was obtained. Some insoluble portion was filtered off, and then, Me_3_SiCl (24.5 g, 225 mmol) was added to the filtrate. Stirring the solution overnight led to the deposition of a purple solid. The solid was isolated via filtration and washed with THF (10 mL) and hexane (10 mL). The isolated solid (6.60 g) was placed in a large vial (~70 mL size) and CH_2_Cl_2_ (53 g) was added to dissolve the solid. When the vial was placed inside a closed chamber (~250 mL size) containing methylcyclohexane (~15 mL), CH_2_Cl_2_ was slowly evaporated and purple crystals were deposited within 24 h, which were isolated by decantation (4.55 g, 69%).

### 3.3. [(CH_3_CN)_4_CrCl_2_]^+^[B(C_6_F_5_)_4_]^−^

A solution of [(CH_3_CN)_4_Ag]^+^[B(C_6_F_5_)_4_]^−^ (7.789 g, 8.189 mmol) in acetonitrile (17.3 g) was added to a suspension of [CrCl_2_(μ-Cl)(thf)_2_]_2_ (2.478 g, 8.189 mmol) in acetonitrile (22.6 g). After stirring overnight at 60 °C, precipitated AgCl was removed by filtration and an olive-green solution was obtained. The solvent was removed using a vacuum line to obtain an olive-green solid (7.71 g). The isolated solid (20.0 mg) and 9-methylanthracene (20.0 mg) were dissolved in THF-d_8_, and the ^1^H NMR spectrum of the solution was recorded to count the number of CH_3_CN molecules per each Cr atom, which was 4.1. The yield was 97% based on the formula [(CH_3_CN)_4.1_CrCl_2_]^+^[B(C_6_F_5_)_4_]^−^. Single crystals suitable for X-ray crystallography were grown in CH_2_Cl_2_; a vial containing [(CH_3_CN)_4_CrCl_2_]^+^[B(C_6_F_5_)_4_]^−^ (100 mg) in CH_2_Cl_2_ (1.0 g) was placed inside a closed chamber containing methylcyclohexane to deposit crystals.

### 3.4. [iPrN{P(C_6_H_4_-p-Si(nBu)_3_)_2_}_2_CrCl_2_]^+^[B(C_6_F_5_)_4_]^−^

A solution of iPrN{P(C_6_H_4_-*p*-Si(nBu)_3_)_2_}_2_ (9.50 g, 7.78 mmol) in CH_2_Cl_2_ (120 g) was added dropwise to a solution of [(CH_3_CN)_4.1_CrCl_2_]^+^[B(C_6_F_5_)_4_]^−^ (7.55 g, 7.781 mmol) in CH_2_Cl_2_ (46 g). Upon addition, the color of the solution changed immediately from olive-green to bluish-green. After stirring for 2.5 h, the solvent was completely removed using a vacuum line. The residue was dissolved in a minimal amount of methylcyclohexane (~5 mL), and the solvent was removed using a vacuum line. This procedure was repeated once more to remove any residual CH_3_CN and CH_2_Cl_2_ completely. The residue (15.7 g, 100%) was dissolved in methylcyclohexane (141.3 g) to obtain a 10 wt% solution, which was used for ethylene tetramerization.

### 3.5. [{Et(Cl)Al(N(iPr)_2_)_2_}Cr(μ-Cl)]_2_

[CrCl_2_(μ-Cl)(thf)_2_]_2_ (0.312 g, 1.03 mmol) was added to a solution of Li^+^[Et_2_Al(N(iPr)_2_)_2_]^−^ (0.302 g, 1.03 mmol) in toluene (4.5 mL) at −30 °C. The mixture was stirred for 8 h at room temperature. The color of the solution changed from dark brown to deep greenish-blue, and LiCl precipitated. The solvent was removed using a vacuum line, and the residue was dissolved in hexane (30 mL). After removal of LiCl via filtration, the filtrate was concentrated to ~10 mL and allowed to stand at −30 °C for 2 days. Blue crystals were deposited (0.178 g, 45%). A second crop of blue crystals was obtained from the mother liquor (0.028 g, 7%).

### 3.6. X-ray Crystallography

Specimens of suitable quality and size were selected, mounted, and centered in the X-ray beam using a video camera. Reflection data were collected at 100 K on an APEX II CCD area diffractometer (Bruker, Billerica, MA, USA) using graphite-monochromated Mo K–α radiation (λ = 0.7107 Ǻ). The hemisphere of the reflection data was collected as φ and ω scan frames at 0.5° per frame and an exposure time of 10 s per frame. The cell parameters were determined and refined using the SMART program. Data reduction was performed using the SAINT software. The data were corrected for Lorentz and polarization effects. An empirical absorption correction was applied using the SADABS program. The structure was solved by direct methods and refined by the full matrix least-squares method using the SHELXTL package and olex2 program with anisotropic thermal parameters for all non-hydrogen atoms. 

Crystallographic data for [CrCl_2_(μ-Cl)(thf)_2_]_2_ (CCDC# 2044805) that were used in all calculations are as follows: C_8_H_15.5_Cl_3_CrO_2_, *M* = 302.05, triclinic, *a* = 6.8070(8), *b* = 9.3170(11), *c* = 10.3192(13) Å, α = 108.070(5)°, β = 96.448(5)°, γ = 94.719(5), *V* = 613.47(13) Å^3^, space group *P*-1, *Z* = 2, and 2285 unique (R(int) = 0.0505). The final *wR*_2_ was 0.1251 (*I* > 2σ(*I*)). 

Crystallographic data for [(CH_3_CN)_4_CrCl_2_]^+^[B(C_6_F_5_)_4_]^−^·2(CH_2_Cl_2_) (CCDC# 2044806) that were used in all calculations are as follows: C_34.5_H_17_BCl_7_CrF_20_N_4_, *M* = 1178.48, monoclinic, *a* = 12.9472(4), *b* = 8.2694(3), *c* = 21.7555(8) Å, β = 99.8523(18)°, *V* = 2294.91(14) Å^3^, space group *P*2/*c*, *Z* = 2, and 4409 unique (R(int) = 0.0285). The final *wR*_2_ was 0.1594 (*I* > 2σ(*I*)). 

Crystallographic data for [{Et(Cl)Al(N(iPr)_2_)_2_}Cr(μ-Cl)]_2_ (CCDC# 2044807) that were used in all calculations are as follows: C_28_H_66_Al_2_Cl_4_Cr_2_N_4_, *M* = 758.60, monoclinic, *a* = 10.2937(2), *b* = 12.8707(3), *c* = 14.7815(3) Å, β = 96.5009(18)°, *V* = 1945.77(7) Å^3^, space group *P*2_1_/n, *Z* = 2, and 3675 unique (R(int) = 0. 1527). The final *wR*_2_ was 0.0909 (*I* > 2σ(*I*)).

## 4. Conclusions

The commonly used CrCl_3_(thf)_3_ is impure obtaining as an irregular solid with no possibility of recrystallization. Another form of the THF adduct of CrCl_3_ (i.e., [CrCl_2_(μ-Cl)(thf)_2_]_2_) was facilely prepared in this work. The structure of [CrCl_2_(μ-Cl)(thf)_2_]_2_ determined by X-ray crystallography agreed well with the XRD patterns and the C content of the bulk. Using well-defined [CrCl_2_(μ-Cl)(thf)_2_]_2_ instead of the impure CrCl_3_(thf)_3_ as a starting material, the activity of the ethylene tetramerization catalyst [iPrN{P(C_6_H_4_-*p*-Si(nBu)_3_)_2_}_2_CrCl_2_]^+^[B(C_6_F_5_)_4_]^−^ was consistent and extremely high (6600 kg/g-Cr/h), and some Cr complexes (e.g., [(CH_3_CN)_4_CrCl_2_]^+^[B(C_6_F_5_)_4_]^−^ and [{Et(Cl)Al(N(iPr)_2_)_2_}Cr(μ-Cl)]_2_) whose structure determination had failed previously could be isolated as high-quality crystals, enabling structure determination by X-ray crystallography. We strongly suggest the use of well-defined [CrCl_2_(μ-Cl)(thf)_2_]_2_ instead of the impure CrCl_3_(thf)_3_ as a Cr source in the synthesis of organometallic and coordination compounds of Cr.

## 5. Patents

A patent was applied on this study (Ajou University, Chromium Compound and Method for Preparing the Same. Kr 10-2020-0052494, 29 April 2020).

## Data Availability

The data that support the findings of this study are available from the corresponding author upon reasonable request.

## Sample Availability

Samples of [CrCl_2_(μ-Cl)(thf)_2_]_2_ are available from the Precious Catalysts Inc.; cglee@s-pci.com (C.G.L.).

## References

[1] Ambrose K., Murphy J.N., Kozak C.M. (2020). Chromium Diamino-bis(phenolate) Complexes as Catalysts for the Ring-Opening Copolymerization of Cyclohexene Oxide and Carbon Dioxide. Inorg. Chem..

[2] Jiménez J.-R., Poncet M., Doistau B., Besnard C., Piguet C. (2020). Luminescent polypyridyl heteroleptic CrIII complexes with high quantum yields and long excited state lifetimes. Dalton Trans..

[3] Baek J.W., Hyun Y.B., Lee H.J., Lee J.C., Bae S.M., Seo Y.H., Lee D.G., Lee B.Y. (2020). Selective Trimerization of α-Olefins with Immobilized Chromium Catalyst for Lubricant Base Oils. Catalysts.

[4] Song T., Tao X., Tong X., Liu N., Gao W., Mu X., Mu Y. (2019). Synthesis and characterization of chromium complexes 2-Me_4_CpC_6_H_4_CH_2_(R)NHCrCl_2_ and their catalytic properties in ethylene homo- and co-polymerization. Dalton Trans..

[5] Sydora O.L., Hart R.T., Eckert N.A., Martinez Baez E., Clark A.E., Benmore C.J. (2018). A homoleptic chromium(iii) carboxylate. Dalton Trans..

[6] Olafsen B.E., Crescenzo G.V., Moisey L.P., Patrick B.O., Smith K.M. (2018). Photolytic Reactivity of Organometallic Chromium Bipyridine Complexes. Inorg. Chem..

[7] Ojelere O., Graf D., Mathur S. (2019). Molecularly Engineered Lithium–Chromium Alkoxide for Selective Synthesis of LiCrO_2_ and Li_2_CrO_4_ Nanomaterials. Inorganics.

[8] Förster C., Dorn M., Reuter T., Otto S., Davarci G., Reich T., Carrella L., Rentschler E., Heinze K. (2018). Ddpd as Expanded Terpyridine: Dramatic Effects of Symmetry and Electronic Properties in First Row Transition Metal Complexes. Inorganics.

[9] Zhao R., Ma J., Zhang H., Huang J. (2017). Synthesis and Characterization of Constrained Geometry Oxygen and Sulphur Functionalized Cyclopentadienylchromium Complexes and Their Use in Catalysis for Olefin Polymerization. Molecules.

[10] Pei L., Tang Y., Gao H. (2016). Homo- and Copolymerization of Ethylene and Norbornene with Anilido–Imine Chromium Catalysts. Polymers.

[11] Herwig W., Zeiss H. (1958). Notes: Chromium Trichloride Tetrahydrofuranate. J. Org. Chem..

[12] Mastalir M., Glatz M., Stöger B., Weil M., Pittenauer E., Allmaier G., Kirchner K. (2017). Synthesis, characterization and reactivity of vanadium, chromium, and manganese PNP pincer complexes. Inorg. Chim. Acta.

[13] Köhn R.D., Coxon A.G.N., Hawkins C.R., Smith D., Mihan S., Schuhen K., Schiendorfer M., Kociok-Köhn G. (2014). Selective trimerisation and polymerisation of ethylene: Halogenated chromium triazacyclohexane complexes as probes for an internal ‘halogen effect’. Polyhedron.

[14] Heisig G.B., Hedin H. (1946). Anhydrous Chromium(III) Chloride. Inorg. Synth..

[15] Vavoulis A., Austin T.E., Tyree S.Y. (1960). Anhydrous Chromium(III) Chloride. Inorg. Synth..

[16] Shamir J. (1989). New synthesis of chromium trichloride tetrahydrofuranate. Inorg. Chim. Acta.

[17] Pray A.R. (1957). Anhydrous metal chlorides. Inorg. Synth..

[18] House D.A., Wang J., Nieuwenhuyzen M. (1995). The synthesis and X-ray structure of *trans*-[CrCl_2_(nPrNH_2_)_4_]BF_4_·H_2_O and the thermal and Hg^2+^-assisted chloride release kinetics from some *trans*-[CrCl_2_(N)_4_]^+^ complexes. Inorg. Chim. Acta.

[19] So J.H., Boudjouk P. (1990). A convenient synthesis of solvated and unsolvated anhydrous metal chlorides via dehydration of metal chloride hydrates with trimethylchlorosilane. Inorg. Chem..

[20] Jeon J.Y., Park J.H., Park D.S., Park S.Y., Lee C.S., Go M.J., Lee J., Lee B.Y. (2014). Concerning the chromium precursor CrCl_3_(thf)_3_. Inorg. Chem. Commun..

[21] Sydora O.L., Knudsen R.D., Baralt E.J. (2013). Preparation of an Olefin Oligomerization Catalyst. U.S. Patent.

[22] Bollmann A., Blann K., Dixon J.T., Hess F.M., Killian E., Maumela H., McGuinness D.S., Morgan D.H., Neveling A., Otto S. (2004). Ethylene Tetramerization:  A New Route to Produce 1-Octene in Exceptionally High Selectivities. J. Am. Chem. Soc..

[23] Overett M.J., Blann K., Bollmann A., Dixon J.T., Haasbroek D., Killian E., Maumela H., McGuinness D.S., Morgan D.H. (2005). Mechanistic Investigations of the Ethylene Tetramerisation Reaction. J. Am. Chem. Soc..

[24] Kuhlmann S., Blann K., Bollmann A., Dixon J.T., Killian E., Maumela M.C., Maumela H., Morgan D.H., Prétorius M., Taccardi N. (2007). N-substituted diphosphinoamines: Toward rational ligand design for the efficient tetramerization of ethylene. J. Catal..

[25] Boelter S.D., Davies D.R., Margl P., Milbrandt K.A., Mort D., Vanchura B.A., Wilson D.R., Wiltzius M., Rosen M.S., Klosin J. (2020). Phospholane-Based Ligands for Chromium-Catalyzed Ethylene Tri- and Tetramerization. Organometallics.

[26] Hirscher N.A., Perez Sierra D., Agapie T. (2019). Robust Chromium Precursors for Catalysis: Isolation and Structure of a Single-Component Ethylene Tetramerization Precatalyst. J. Am. Chem. Soc..

[27] Lee H., Hong S.H. (2018). Polyhedral oligomeric silsesquioxane-conjugated bis(diphenylphosphino)amine ligand for chromium(III) catalyzed ethylene trimerization and tetramerization. Appl. Catal. A. Gen..

[28] Newland R.J., Smith A., Smith D.M., Fey N., Hanton M.J., Mansell S.M. (2018). Accessing Alkyl- and Alkenylcyclopentanes from Cr-Catalyzed Ethylene Oligomerization Using 2-Phosphinophosphinine Ligands. Organometallics.

[29] Nobbs J.D., Tomov A.K., Young C.T., White A.J.P., Britovsek G.J.P. (2018). From alternating to selective distributions in chromium-catalysed ethylene oligomerisation with asymmetric BIMA ligands. Catal. Sci. Technol..

[30] Alam F., Zhang L., Wei W., Wang J., Chen Y., Dong C., Jiang T. (2018). Catalytic Systems Based on Chromium(III) Silylated-Diphosphinoamines for Selective Ethylene Tri-/Tetramerization. ACS Catal..

[31] Höhne M., Peulecke N., Konieczny K., Müller B.H., Rosenthal U. (2017). Chromium-Catalyzed Highly Selective Oligomerization of Ethene to 1-Hexene with N,N-Bis[chloro(aryl)phosphino]amine Ligands. ChemCatChem.

[32] Lifschitz A.M., Hirscher N.A., Lee H.B., Buss J.A., Agapie T. (2017). Ethylene Tetramerization Catalysis: Effects of Aluminum-Induced Isomerization of PNP to PPN Ligands. Organometallics.

[33] Tomov A.K., Nobbs J.D., Chirinos J.J., Saini P.K., Malinowski R., Ho S.K.Y., Young C.T., McGuinness D.S., White A.J.P., Elsegood M.R.J. (2017). Alternating α-Olefin Distributions via Single and Double Insertions in Chromium-Catalyzed Ethylene Oligomerization. Organometallics.

[34] Zhang L., Meng X., Chen Y., Cao C., Jiang T. (2017). Chromium-Based Ethylene Tetramerization Catalysts Supported by Silicon-Bridged Diphosphine Ligands: Further Combination of High Activity and Selectivity. ChemCatChem.

[35] Kim E.H., Lee H.M., Jeong M.S., Ryu J.Y., Lee J., Lee B.Y. (2017). Methylaluminoxane-Free Chromium Catalytic System for Ethylene Tetramerization. ACS Omega.

[36] Kim T.H., Lee H.M., Park H.S., Kim S.D., Kwon S.J., Tahara A., Nagashima H., Lee B.Y. (2019). MAO-free and extremely active catalytic system for ethylene tetramerization. Appl. Organomet. Chem..

[37] Park H.S., Kim T.H., Baek J.W., Lee H.J., Kim T.J., Ryu J.Y., Lee J., Lee B.Y. (2019). Extremely Active Ethylene Tetramerization Catalyst Avoiding the Use of Methylaluminoxane: [iPrN{P(C_6_H_4_-*p*-SiR_3_)_2_}_2_CrCl_2_]^+^[B(C_6_F_5_)_4_]^−^. ChemCatChem.

[38] Ewart S.W., Kolthammer B.W.S., Smith D.M., Hanton M.J., Dixon J.T., Morgan D.H., Debod H., Gabrielli W.F., Evans S.J. (2010). Oligomerization of Olefinic Compounds in the Presence of an Activated Oligomerization Catalyst. U.S. Patent.

[39] Stennett T.E., Haddow M.F., Wass D.F. (2012). Avoiding MAO: Alternative Activation Methods in Selective Ethylene Oligomerization. Organometallics.

[40] McGuinness D.S., Rucklidge A.J., Tooze R.P., Slawin A.M.Z. (2007). Cocatalyst Influence in Selective Oligomerization:  Effect on Activity, Catalyst Stability, and 1-Hexene/1-Octene Selectivity in the Ethylene Trimerization and Tetramerization Reaction. Organometallics.

[41] McGuinness D.S., Overett M., Tooze R.P., Blann K., Dixon J.T., Slawin A.M.Z. (2007). Ethylene Tri- and Tetramerization with Borate Cocatalysts:  Effects on Activity, Selectivity, and Catalyst Degradation Pathways. Organometallics.

[42] Hirscher N.A., Labinger J.A., Agapie T. (2019). Isotopic labelling in ethylene oligomerization: Addressing the issue of 1-octene vs. 1-hexene selectivity. Dalton Trans..

[43] Hirscher N.A., Agapie T. (2017). Stoichiometrically Activated Catalysts for Ethylene Tetramerization using Diphosphinoamine-Ligated Cr Tris(hydrocarbyl) Complexes. Organometallics.

[44] Albert Cotton F., Duraj S.A., Powell G.L., Roth W.J. (1986). Comparative structural studies of the first row early transition metal(III) chloride tetrahydrofuran solvates. Inorg. Chim. Acta.

[45] Köhn R.D., Coxon A.G.N., Chunawat S., Heron C., Mihan S., Lyall C.L., Reeksting S.B., Kociok-Köhn G. (2020). Triazacyclohexane chromium triflate complexes as precursors for the catalytic selective olefin trimerisation and its investigation by mass spectrometry. Polyhedron.

[46] Kern R.J. (1962). Tetrahydrofuran complexes of transition metal chlorides. J. Inorg. Nucl. Chem..

[47] Dixon J.T., Green M.J., Hess F.M., Morgan D.H. (2004). Advances in selective ethylene trimerization—A critical overview. J. Organomet. Chem..

[48] Jeon J.Y., Park D.S., Lee D.H., Eo S.C., Park S.Y., Jeong M.S., Kang Y.Y., Lee J., Lee B.Y. (2015). A chromium precursor for the Phillips ethylene trimerization catalyst: (2-ethylhexanoate)_2_CrOH. Dalton Trans..

